# CT-based deep learning model for the prediction of DNA mismatch repair deficient colorectal cancer: a diagnostic study

**DOI:** 10.1186/s12967-023-04023-8

**Published:** 2023-03-22

**Authors:** Wuteng Cao, Huabin Hu, Jirui Guo, Qiyuan Qin, Yanbang Lian, Jiao Li, Qianyu Wu, Junhong Chen, Xinhua Wang, Yanhong Deng

**Affiliations:** 1 Department of Radiology, The Sixth Affiliated Hospital, Sun Yat-Sen University, Guangzhou, 510655 Guangdong China; 2 Guangdong Provincial Key Laboratory of Colorectal and Pelvic Floor Diseases, Guangdong Research Institute of Gastroenterology, The Sixth Affiliated Hospital, Sun Yat-Sen University, Guangzhou, 510655 Guangdong China; 3 Department of Medical Oncology, The Sixth Affiliated Hospital, Sun Yat-Sen University, Guangzhou, 510655 Guangdong China; 4 Department of Colorectal Surgery, Department of General Surgery, The Sixth Affiliated Hospital, Sun Yat-Sen University, Guangzhou, 510655 Guangdong China; 5 Department of Radiology, The First Affiliated Hospital of Zhengzhou University, Zhengzhou, 450052 Henan China; 6 School of Public Health (Shenzhen), Shenzhen Campus of Sun Yat-Sen University, Shenzhen, 518107 Guangdong China

**Keywords:** DNA mismatch repair, Deep learning, Colorectal cancer, Computed Tomography, ResNet101

## Abstract

**Background:**

Stratification of DNA mismatch repair (MMR) status in patients with colorectal cancer (CRC) enables individual clinical treatment decision making. The present study aimed to develop and validate a deep learning (DL) model based on the pre-treatment CT images for predicting MMR status in CRC.

**Methods:**

1812 eligible participants (training cohort: n = 1124; internal validation cohort: n = 482; external validation cohort: n = 206) with CRC were enrolled from two institutions. All pretherapeutic CT images from three dimensions were trained by the ResNet101, then integrated by Gaussian process regression (GPR) to develop a full-automatic DL model for MMR status prediction. The predictive performance of the DL model was evaluated using the area under the receiver operating characteristic curve (AUC) and then tested in the internal and external validation cohorts. Additionally, the participants from institution 1 were sub-grouped by various clinical factors for subgroup analysis, then the predictive performance of the DL model for identifying MMR status between participants in different groups were compared.

**Results:**

The full-automatic DL model was established in the training cohort to stratify the MMR status, which presented promising discriminative ability with the AUCs of 0.986 (95% CI 0.971–1.000) in the internal validation cohort and 0.915 (95% CI 0.870–0.960) in the external validation cohort. In addition, the subgroup analysis based on the thickness of CT images, clinical T and N stages, gender, the longest diameter, and the location of tumors revealed that the DL model showed similar satisfying prediction performance.

**Conclusions:**

The DL model may potentially serve as a noninvasive tool to facilitate the pre-treatment individualized prediction of MMR status in patients with CRC, which could promote the personalized clinical-making decision.

**Supplementary Information:**

The online version contains supplementary material available at 10.1186/s12967-023-04023-8.

## Background

Colorectal cancer (CRC) is the third most commonly diagnosed malignancy in the world and the second highest rate of increasing incidence among all gastrointestinal tumors [1, 2]. Patients with DNA mismatch repair deficient (dMMR)/microsatellite instability-high (MSI-H) CRC have a short overall survival (OS) and obtain no benefit from adjuvant chemotherapy [3–5]. More importantly, recent studies have demonstrated dMMR/MSI-H is a predictive biomarker for immunotherapy, because dMMR/MSI-H CRCs are associated with a higher mutational burden tumor neoantigen load, and dense immune cell infiltration [6, 7]. In addition, immunotherapy in patients with advanced CRC harboring dMMR/MSI has been approved by the United State Food and Drug Administration (FDA).

The National Comprehensive Cancer Network (NCCN) and the European Society for Medical Oncology (ESMO) guidelines both recommended that all patients with CRC be tested for microsatellite instability, a hypermutable phenotype caused by defects in DNA mismatch repair, which facilitated individualized clinical making-decisions, then maximized the benefits for CRC patients [8–10]. Current testing for dMMR/MSI include PCR-based assay for microsatellite markers and immunohistochemical analysis for MMR protein expression [11]. While these existed approaches face distinct drawbacks. First, routine MSI testing using IHC or PCR is not commonly performed on account of tedious procedures and the heavy financial burden [12, 13]. In addition, the procedure of sampling is invasive linked to potentially complications, which limits the dynamic monitoring of biological characteristics and histopathological changes of tumors [14]. Furthermore, the accuracy of conventional biopsy specimens will be influenced by sampling errors, such as insufficient or inappropriate tissue sampling because of tumor heterogeneity. Therefore, a noninvasive and accurate method is highly desirable for pretherapeutic prediction of MMR status, to help better stratify CRC patients before individualized clinical making-decision.

Recently, artificial intelligence (AI) algorithms, particularly deep learning, have shown outstanding performance in medical image processing, advancing the field forward at a rapid pace [15]. A typical approach of deep learning termed convolutional neural network (CNN) has been reported to act as an alternative tool to tackle complex medical issues efficiently and effectively and achieve satisfying performance in many diseases such as breast cancer, pulmonary nodules, gastrointestinal cancer and CRC [16–19]. Recent studies have proposed deep learning approaches for predicting MMR status based on hematoxylin and eosin histological images in CRC patients [20–22]. These studies reported a moderate predictive performance, which suggested that it is reasonable to speculate that deep learning approaches can achieve improved performance for pretherapeutic prediction of MMR status in CRC. However, few studies based on deep learning focus on routine and noninvasive computed tomography (CT) images.

In the present study, we aim to develop and validate a deep learning model based on pretherapeutic CT images for predicting MMR status in CRC. We hypothesized that DL model could make it easier and more precisive to stratify MMR status, which could promote the personalized clinical-making decision.

## Methods

### Study participants

The retrospective study was approved by Ethics Committees of the two participating institutions and the informed consent requirement was waived due to its retrospective nature. Consecutive patients with histologically confirmed primary CRC between March 2012 and March 2020 were retrospectively reviewed from two medical institutions: the Sixth Affiliated Hospital of Sun Yat-sen University (Guangzhou, China, institution 1) and the First Affiliated Hospital of Zhengzhou University (Zhengzhou, China, institution 2). Our inclusion criteria were patients with (i) pathologically confirmed primary CRC, (ii) available test results for MMR status, (iii) pretherapeutic contrast-enhanced abdominopelvic CT images within 2 weeks before surgery, (iv) available complete clinicopathological data. The exclusion criteria were as follows: (i) receiving any therapy before CT examination, (ii) lacking MMR test, (iii) incomplete clinicopathological data, (iv) the interval between CT examinations and surgery over 2 weeks, and (v) inoperability or refusal of operation.

A total of 1812 eligible patients were enrolled in this study. The patients from institution 1 were divided into a training cohort (n = 1124, March 2012 to March 2018) and an internal validation cohort (n = 482, May 2018 to March 2020) by time. The 206 participants from institution 2 were assigned to an external cohort. It’s noted that no data from the same patients in the training and external cohorts. The detail of the recruitment pathway was presented in Additional file 1: Fig. S1.

### Clinicopathological characteristics

**Fig. 1:**
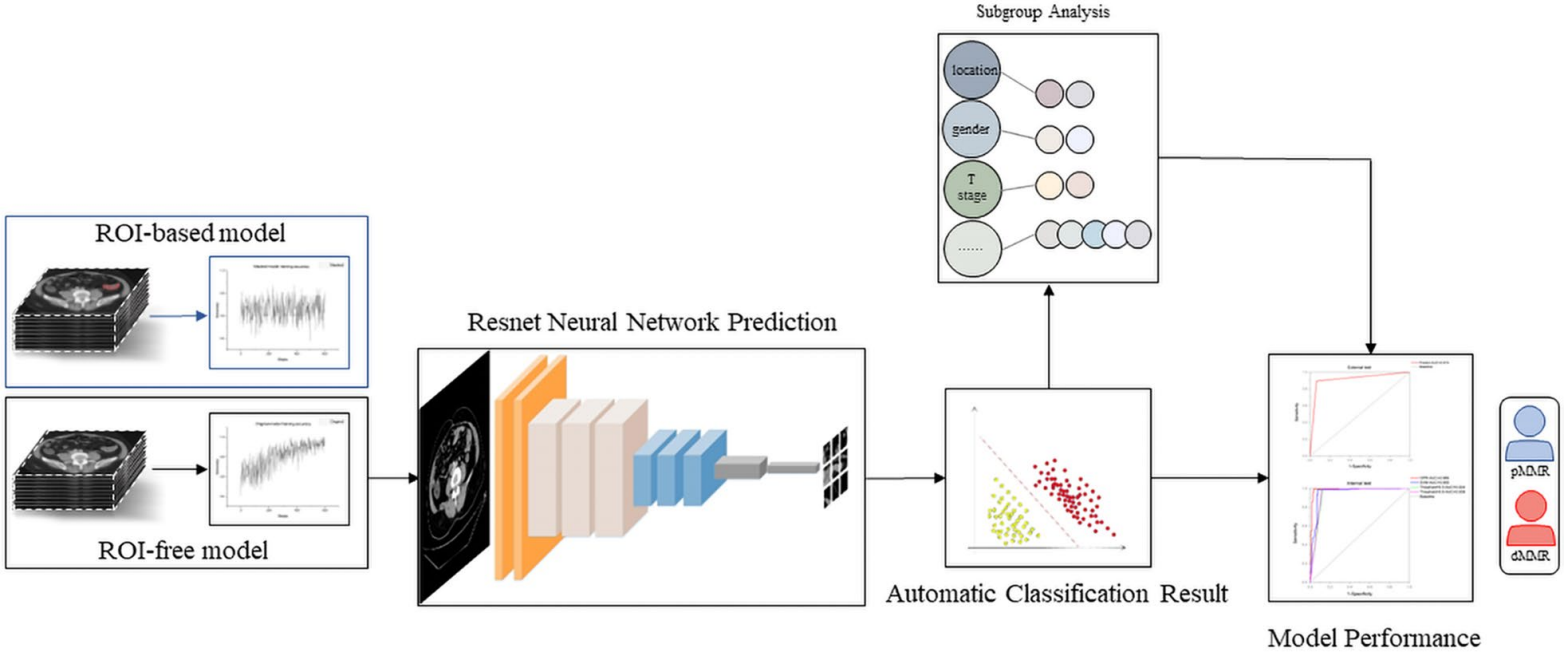
The overall experimental design in the study

The baseline clinicopathological parameters of patients from institution 1, including gender, age, the levels of serum tumor markers, such as carcinoembryonic antigen (CEA), cancer antigen 199 (CA199), cancer antigen 125 (CA125) and cancer antigen 153 (CA153), the clinical T and N stage, the longest diameter, and the location of tumors, were retrospectively reviewed and recorded from the medical record archives. Additionally, the thickness of CT images was also recorded. The overall experimental design is shown in Fig. 1.

### Identification of MMR status

Immunohistochemistry (IHC) analysis of mismatch repair (MMR) proteins expression–Formalin-fixed paraffin-embedded (FFPE) tumors were examined the loss of MMR proteins (MLH1, MSH2, MSH6, and PMS2) expression. MMR protein loss is defined as the absence of nuclear staining in neoplastic cells but positive nuclear staining in lymphocytes and normal adjacent colonic epithelium. Primary monoclonal antibodies against MLH1, MSH2, MSH6, and PMS2 were applied. MMR status was determined locally by IHC analysis and tumors displaying loss of at least one of four MMR proteins can be considered as deficient mismatch repair (dMMR), whereas those with intact MMR proteins can be classified as proficient mismatch repair (pMMR).

### CT image acquisition

All patients underwent contrast-enhanced CT scans covered the abdominal and pelvic region. CT images were obtained using three CT scanners from two institutions. For institution 1, patients were examined using OPTIMA CT660 (GE Medical Systems, Milwaukee, WI, United States) or AQUILION ONE (TOSHIBA Medical Systems, Japan) scanner. For institution 2, 64-row multidetector device (Discovery CT750HD, GE Medical Systems, Waukesha, WI, United States) CT scanner was used to perform abdominopelvic CT scans. The acquisition parameters of the two institutions were as follows: tube voltage of 120 kV; tube current of 150–550 mA; pitch of 0.97 to 0.99; reconstruction section thickness of 1.25 mm and 5 mm. The contrast agents at the dose of 1.2–1.5 mL/kg weight were injected at a speed of 2.5–3 mL/s with a high-pressure pump syringe. Arterial phase was obtained after 25–30 s of delay after intravenous injection of contrast material, and portal venous phase was performed after 55–70 s of delay. The representative CT and immunohistochemistry images of different MMR statuses were shown in Additional file 5: Material S1.

### Preliminary experiment

All CT image data were derived from the Picture Archiving and Communication System (PACS) then converted into a unified Communications in Medicine (DICOM) format and stored as Nifti format on a case-by-case basis for further analysis. A preliminary experiment was conducted to determine the performance of region of interest (ROI)-based labeling approaches (method 1) for modeling versus ROI-free analysis (method 2) in prediction of MMR status for CRC patients, then that approach with better predictive performance would be selected as the final data processing method in the present study. The preliminary experiment consisted of 100 participants randomly selected from institution 1, including 50 patients with dMMR and 50 with pMMR.

For the method 1, three-dimensional manual segmentation of the tumor ROI was performed on the portal venous phase CT images by one radiologist with 10 years of experience with CRC diagnosis, using the free open-source software ITK-SNAP software (version 2.2.0, http://www.itksnap.org), with careful exclusion of pericolonic fat and mesentery air. Note that each segmentation was validated by a senior radiologist, who had 20 years of experience. Next, the obtained images were segmented and stored as PNG image files with the same resolution based on the axial direction, then divided into training group and test group. For the method 2, we directly processed the images of the original Nifti format files without delineating the ROIs, and these images also were converted and stored as PNG image files, then processed by the same approach as described in method 1 above. The images processed by the above two methods were saved separately as independent datasets. Whereafter, a classification neural network architecture (ResNet101) was applied to train the two independent datasets within the same development environment, meanwhile, we set the maximum training epochs to 100 epochs in both experiments. Finally, we compared the accuracy of the two models on the validation set, the model with better predictive performance would be identified, the corresponding data processing method in this study was finalized.

The preliminary experiment suggested that the model based on ROI labeling approach (method 1) showed inferior accuracy in predicting MMR status, with the accuracy of 73% while the other model based on fully automatic deep learning analysis (model 2) yielded an accuracy of more than 90%. Therefore, ROI-free analysis based on ResNet101 was identified as the final data processing method in the present study.

### MMRnet development

On the basis of the preliminary experiment, we selected the method 2 (ROI-free) to process CT images data and construct the predictive model named “MMRnet”. It’s noted that all 1812 enrolled patients received abdominopelvic contrast-enhanced CT, which covered the whole tumors of colon or rectum. All CT images data were derived from the PACS, then converted into a unified Communications in Medicine (DICOM) format and stored as Nifti format on a case-by-case basis as similar as the preliminary experiment. By introducing the opencv-python package and writing related packages, the Nifti format files were divided into PNG image files with the same resolution in the axial direction and divided into training files and test files based on the axial direction.

The ResNet101 architecture, one of Resnet model, was utilized to train the pre-therapeutic CT imaging data and build the network to identify the MMR status, which contains one 7*7 convolutional layer, one max pooling layer, 33 bottleneck and one fully connected layer. Each bottleneck contains one 1 × 1 convolutional layer, one 3 × 3 convolutional layer and one 1 × 1 convolutional layer. The final fully connected layer output prediction. Images were transformed into matrices and input to the ResNet101 neural networks. During the training process, we used a cross-entropy loss function and Stochastic Gradient Descent (SGD). We set the initial learning rate to 0.001, the weight decay to 0.005, and the batch size to 128. The final epoch of model training was 100. In this study, we analyzed multi-axial CT images to develop a 3D predictive model, all CT images of the training cohort were automatically recognized and interpreted by ResNet101 with Pytorch (version 1.1.0), and the corresponding information of different axial CT images was obtained. Note that the mapping relationship between different axial images is considerably complex, and it is difficult to fuse complex information by using a general linear model. In order to take full advantage of all image information, we applied the Gaussian process regression (GPR) model to fuse the information of different axial images for automatic interpretation results of CT images. The GPR models are constructed from classical statistical models by replacing latent functions of parametric form by random processes with Gaussian prior, which is widely used in regression and classification tasks [23]. Additionally, the model can utilize prior prediction knowledge to provide predictive results. For regression tasks of large dataset, the GPR can reduce computational complexity [24]. The GPR model was used to perform regression analysis on all CT image interpretation results, which improved the accuracy of fusion results to a certain extent.

**Fig. 2:**
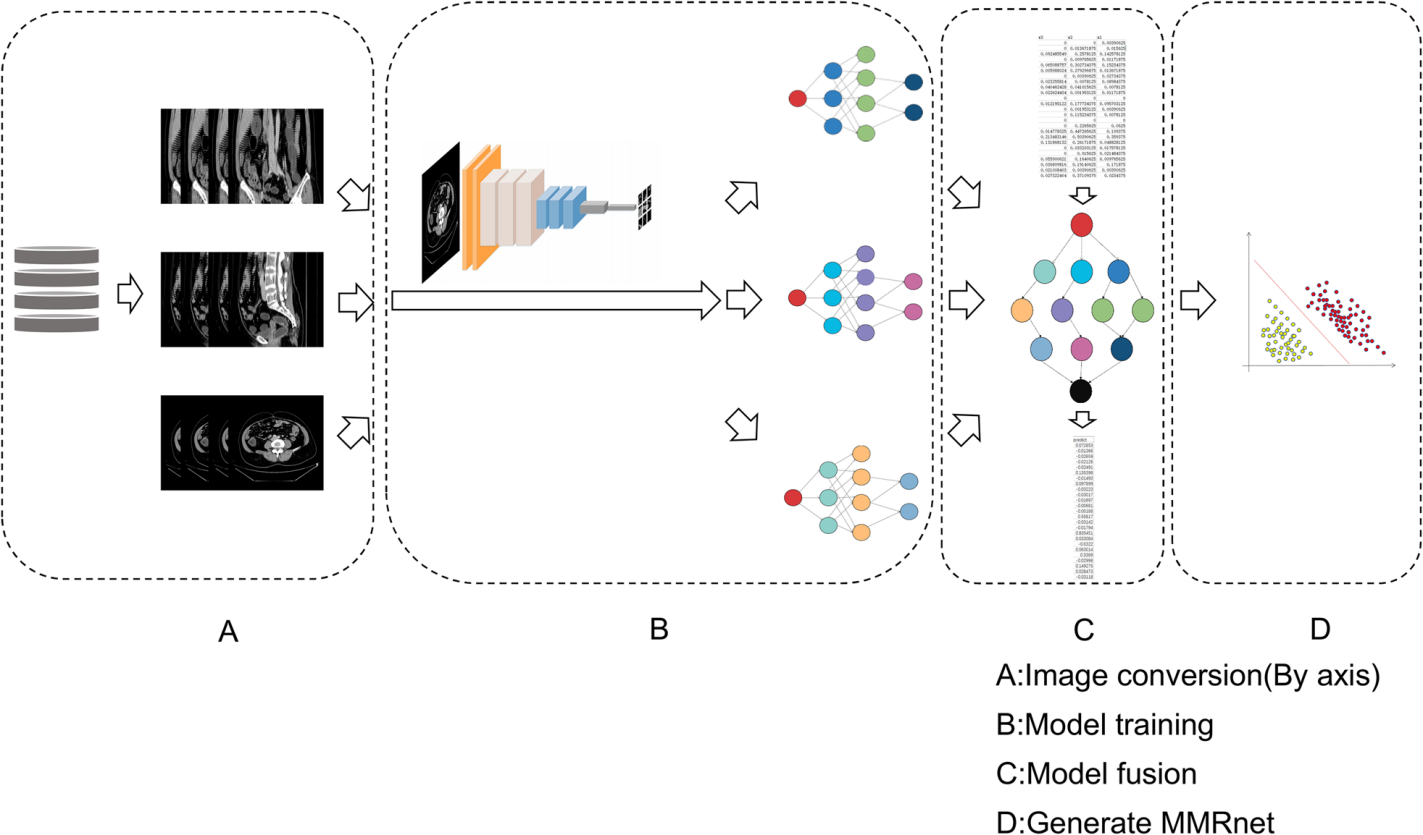
Overview of our deep learning system. The system consists of four steps: the conversion of CT images based on three axis, model training based on Resnet101, model fusion by Gaussian regression and the generation of MMRnet

The training model performance was evaluated using multi-fold cross-validation. The squared exponential function was finally set as the model kernel function:

$$cov\left({x}_{i},{x}_{j}\right)=\mathrm{exp}(-\frac{{({x}_{i}-{x}_{j})}^{2}}{2})$$

x_i and x_j represents different image interpretation results. The GPR model was fused with neural network and finally achieved automatic machine diagnosis of MMR status (dMMR = 1 or pMMR = 0). The deep learning model workflow is presented in Fig. 2.

### Predictive performance of the MMRnet

The predictive performance of MMRnet was trained to its optimum in the training cohort and then tested in the two validation cohorts. Receiver operating characteristic curve (ROC) analysis was performed to evaluate the performance of MMRnet. The optimal cutoff threshold was identified by maximizing Youden index (sensitivity + specificity − 1), then the area under the receiver operating characteristic curve (AUC), sensitivity, specificity, accuracy, positive predictive value (PPV), and negative predictive value (NPV) were then calculated with the cutoff of ROC curve identified in the primary cohort, which was also applied to the validation cohorts.

### Ablation experiments

To investigate which networks are suitable for accurate MMR status prediction based on ROI-free analysis, we applied the ResNet101 and the VGG-19 network to construct the prediction model respectively, then compared their predictive performance in terms of the AUC value.

### Statistical analysis

The clinical parameters were compared and analyzed by Student t test for continual variables and Chi-square, Fisher's exact, or Mann–Whitney U tests for categorical variables. Statistical analysis was conducted with R software (version 3.5.0; http://www.Rproject.org), MATLAB (version 2020a; Mathworks, Natick, MA, USA) and MedCalc Software (version 18.2 Belgium). A result was considered to indicate a significant difference with a p value of less than 0.05.

## Results

### Patient characteristics

In the present study, 481 patients with dMMR status determined by the IHC analysis, with prevalence of 24.3% (390/1606) in institution 1 and 44.2% (91/206) in institution 2. Patient characteristics and a comparison between patients with dMMR and pMMR in institution 1 are presented in Table 1. In the training cohort and internal validation cohort, no significant difference was found between the dMMR status and pMMR status groups in terms of gender and the levels of serum CEA, CA199, CA153 (p > 0.05). However, regarding the age, N stage and tumor location, significant differences were observed between the two groups in both cohorts from institution 1.

**Table 1:** Characteristics of patients in institution 1

Variable	Training cohort (n = 1124)			Internal validation cohort (n = 482)		
	dMMR (n = 273)	pMMR (n = 851)	p value	dMMR (n = 117)	pMMR (n = 365)	p value
Age	54.0 (42.8, 64.0)	60.0 (50.0, 68.0)	< 0.001	57.0 (44.7, 65.3)	60.0 (51.0, 67.3)	0.022
Gender			0.762			0.241
Male	169	516		64	224	
Female	104	335		53	141	
cT stage			0.145			0.019
1–2	3	19		3	14	
3	168	550		62	241	
4a	94	243		52	107	
4b	8	39		0	3	
cN stage			< 0.001			0.005
0	69	250		17	89	
1	108	443		66	214	
2	96	158		34	62	
Location			< 0.001			< 0.001
Right	168	284		84	119	
Left	105	567		33	246	
Tumor marker						
CEA (IQR)	12.1 (1.4, 4.5)	80.3 (2.1, 13.0)	0.164	10.6 (1.6, 4.3)	23.7 (2.3, 11.1)	0.241
CA125 (IQR)	19.4 (8.6, 17.8)	26.3 (8.7, 21.8)	0.017	17.1 (8.2, 17.3)	32.6 (9.1, 23.4)	0.120
CA199 (IQR)	150.9 (2.7, 20.2)	536.4 (4.1, 30.1)	0.247	27.0 (2.8, 14.8)	156.4 (3.9, 24.3)	0.243
CA153 (IQR)	10.4 (6.3, 13.0)	10.4 (6.3, 12.2)	0.990	9.1 (6.0, 12,1)	10.4 (6.6, 12.2)	0.202

IQR: interquartile range; dMMR: DNA mismatch repair deficient; pMMR: mismatch repair-proficient; cT stage: baseline clinical tumor stage; cN stage: baseline clinical lymph node stage; CEA: carcinoembryonic antigen; CA199: cancer antigen 199; CA125: cancer antigen 125; CA153: cancer antigen 153

### Predictive performance of the ResNet classification model

**Fig. 3:**
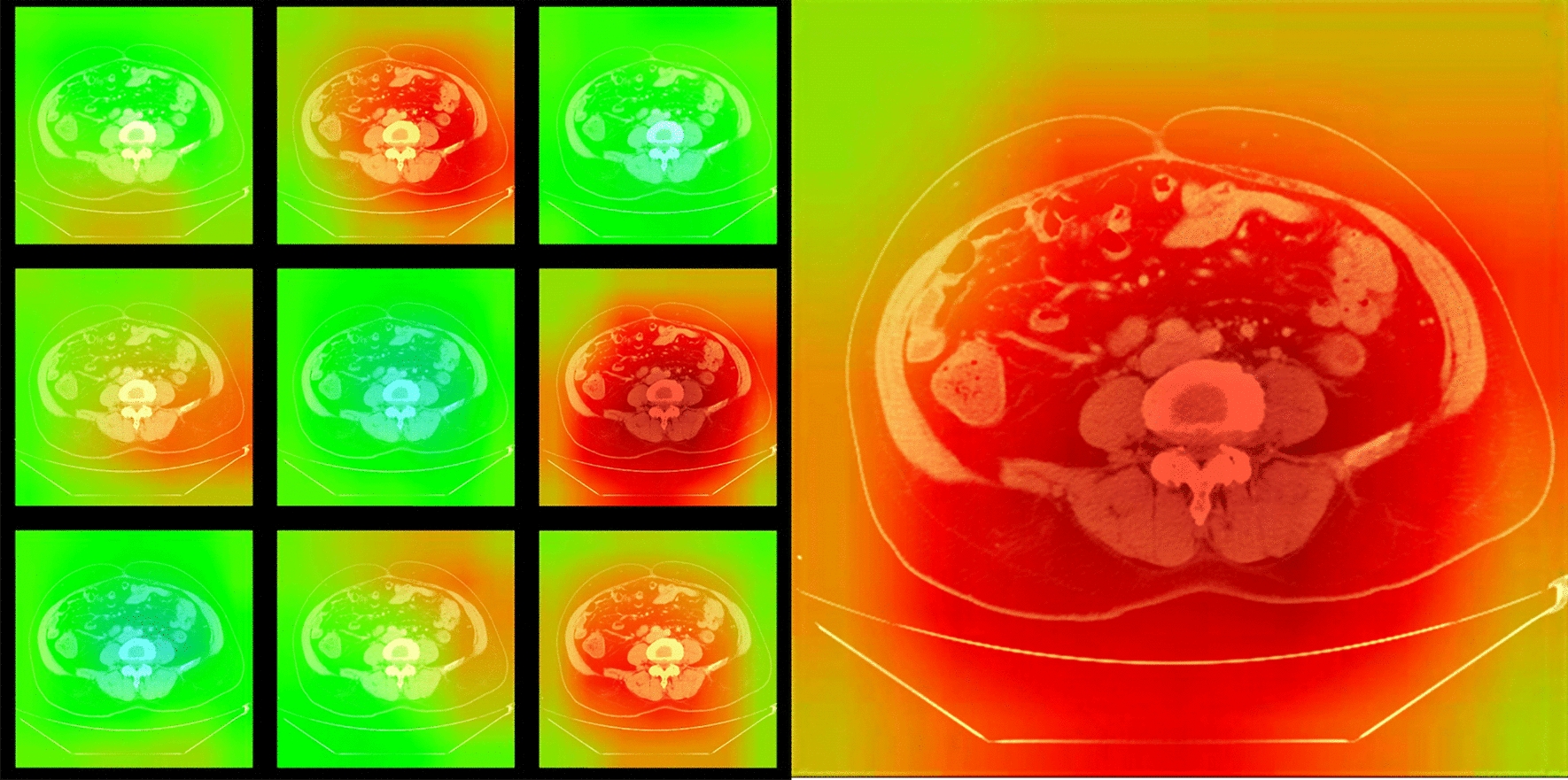
The heatmap. The heatmaps highlight that the region of red color means the higher possibility of the presence of predictive feature, these regions were important in making the diagnosis for the neural network

In comparison of the predictive performance of ResNet101 and VGG-19 in predicting the MMR status, the ResNet101 showed the superior prediction performance, with a higher AUC of 0.997 (95% CI 0.995–1.000, p < 0.001), the corresponding ROC curves were present in Additional file 2: Fig. S2. As demonstrated in the Fig. 3, the heatmap demonstrated that the region of red color means the higher possibility of the presence of predictive features, and these regions were important in making the diagnosis for the neural network, which visualizes the image features corresponding to different convolution layers.

**Fig. 4:**
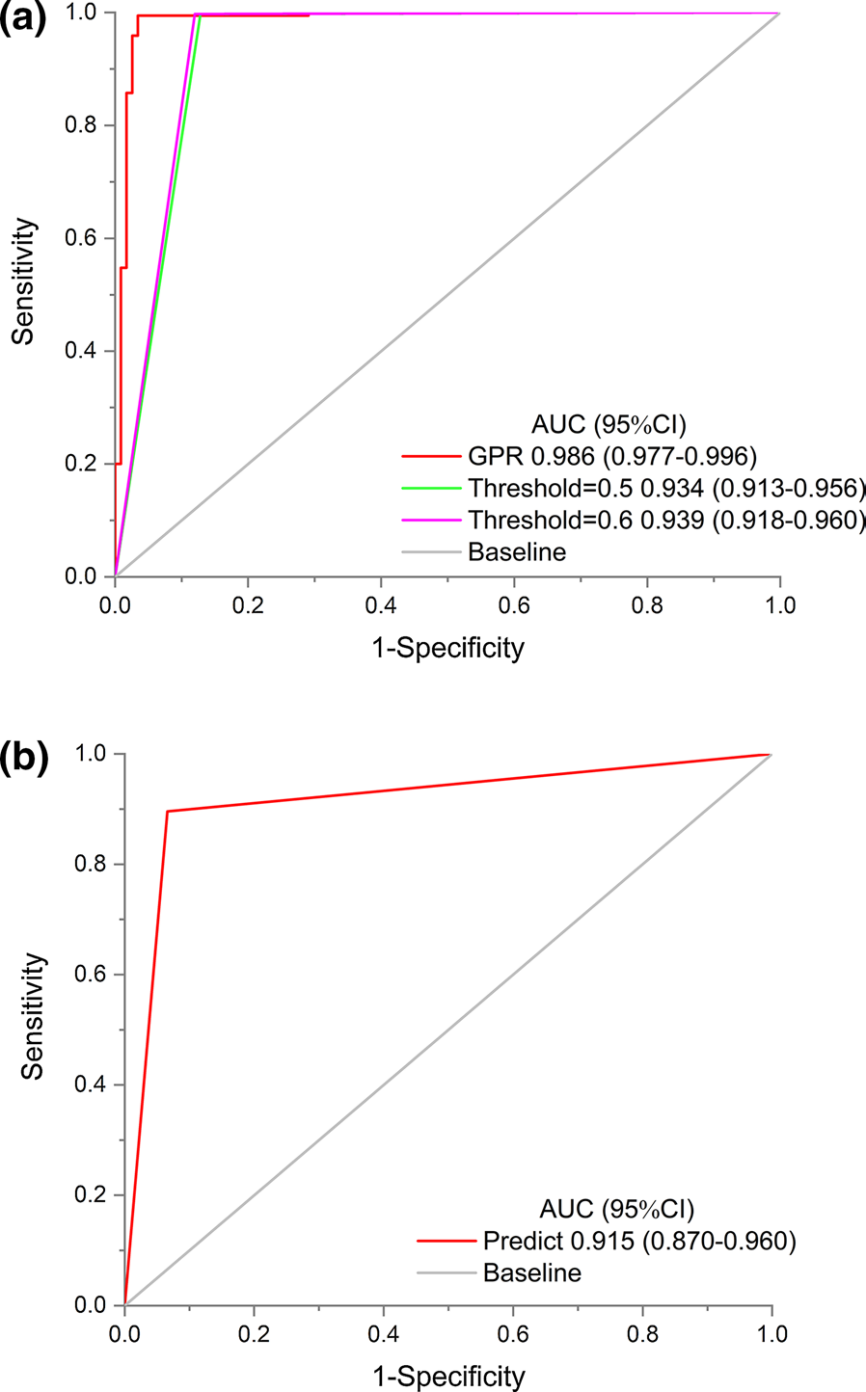
**a** The ROC curves of the MMRnet in the internal validation cohort. The AUCs were reported based on the R software. **b** The ROC curve of the MMRnet in predicting MMR status in the external validation cohort. The AUC was reported

Based on the uniaxial information of CT images, the DL model achieve the AUCs of 0.944, 0.762, 0.984 in the X, Y, Z axis respectively (Additional file 3: Fig. S3). Additionally, in the process of GPR analysis to fuse multi-axial information, we found that the MMRnet model had the optimum prediction performance while the Gaussian process regression squared index was adopted, the details were presented in Additional file 4: Fig. S4. In this condition, for stratifying MMR status, the DL model were developed in the training cohort to automatically classify the MMR status, then tested in two validation cohorts, which achieved promising discriminative ability, with AUCs of 0.986 (95% CI 0.971–1.000) in the internal validation cohort and 0.915 (95% CI 0.870–0.960) in the external validation cohort. The sensitivity, specificity, accuracy, PPV and NPV were also present in Table 2 and Fig. 4.

**Table 2:** Predictive performance of MMRnet in the internal and external validation cohorts

	AUC (95% CI)	Sensitivity	Accuracy	Specificity	PPV	NPV
Internal validation cohort	0.986 (0.971–1.000)	0.995	0.988	0.966	0.989	0.983
External validation cohort	0.915 (0.870–0.960)	0.896	0.913	0.934	0.945	0.876

AUC: area under the receiver operating characteristic curve; PPV: positive predictive value; NPV: negative predictive value

### Subgroup analysis

Subgroups analyses also were performed in addition to main analysis in order to assess prediction performance in different subgroups based on the thickness of CT images, clinical T and N stages, gender, the longest diameter and location of tumor in participants enrolled from institution 1. The subgroup analysis revealed that the MMRnet show similar satisfying prediction performance in all groups, as presented in Additional file 6: Material S2.

## Discussion

We developed a fully automated classifier for stratifying MMR status in 1812 patients with CRC using clinically acquired pretherapeutic CT images from two institutes. It demonstrated promising performance with an AUC of 0.986 in the internal validation cohort; moreover, when further validated in an external validation cohort, it demonstrated robust performance, with an AUC as high as 0.915. It’s noted that the MMRnet based on fully automatic deep learning successfully triaged MMR status in different groups by subgroups analysis. The outperformance of the MMRnet model indicated that CT-based full-automatic deep learning could serve as a noninvasive tool for the pretreatment prediction of MMR status in CRC, further enabling the clinical implementation of computer-aided personalized management for CRC patients.

An architecture of networks was designed by paralleling a max-pooling layer with a center-cropping layer to extract information from different scales. In our study, the Resnet was used for image processing, one of the most popular deep learning models for image analysis, which ease the training of networks that are deeper than those used previously [25]. In addition, we applied the GPR model to fuse the information of different axial images for automatic interpretation results of CT images, which can reduce computational complexity and improve the accuracy of fusion results to a certain extent compared with the general linear model. Our results displayed excellent performance using the DL model to stratify MMR status. Although the underlying mechanism of using deep learning to predict MMR status remains unclear, we hypothesized that this could be related to tumor heterogeneity. After all, the widespread application of deep learning in the non-invasive analysis of tumor heterogeneity in the field of oncology has been demonstrated in many previous studies [26, 27]. Additionally, it is reported that dMMR/MSI-H tumors tend to have distinct morphological patterns such as poor differentiation, mucinous differentiation, histological heterogeneity, infiltrating lymphocytes, and significant Crohn-like reactions at the tumor frontier [28, 29]. These histopathological features imply that the dMMR tumors may be more heterogeneous than pMMR tumors, which could be captured by the DL model. In our study, the DL model achieved excellent performance in MMR status prediction of CRC patients, which was comparable to and even superior to the results of previous studies. It was indicated that deep learning could gain more high-dimensional image information about tumor heterogeneity that cannot be captured by human eyes [20–22]. In addition, CT-based radiomics are predominant in image-based models and have been proposed to preoperative discriminate dMMR and pMMR in colorectal cancer [30–32]. However, tumors were usually segmented manually in majority radiomics researches, which was time-consuming and inevitably caused inter-observer variations. These defects could be avoided by the DL method, and it could adaptively extract features according to the data rather than using predefined features.

In the statistical analysis, we found the age and tumor location present statistical significance in the differentiated dMMR group and pMMR group, indicating that the younger patients and the tumor located in the right colon were more likely to show dMMR status, which was consistent with previous studies [33]. Our study identified that dMMR CRC occurs predominantly in the younger population and on the right side, which may be explained by the fact that the proximal and distal colon have different embryonic origins, leading to distinct biological properties [34, 35]. In the present study, the clinical parameters were not incorporated in the final prediction model, mainly based on the following considerations. Firstly, the fully automatic DL model had outperformed discriminative performance in stratifying MMR status, with an AUC of 0.986 in the internal validation cohort. Meanwhile, the predictive performance was robust in the external validation cohort, which achieved an AUC of 0.915. Hence, we have reason to believe that the DL model developed in the present study could be considered an independent tool to predict the MMR status for CRC patients. In addition, due to the excellent predictive performance of this DL model, the actual predictive efficacy of clinical parameters may be obscured when incorporated into the DL model. Therefore, we tend to construct a relatively simple and feasible DL model rather than a combined model incorporating the predictive clinical parameters. For the clinical parameters, subgroups analyses were performed in addition to the main analysis to assess the prediction performance in different subgroups in this study, and the result revealed that the MMRnet model showed similar satisfying prediction performance in all groups, that indicating the pre-therapeutic CT-based DL model could potentially serve as an alternative approach to predict the MMR status, then help in clinical decision-making for CRC patients.

Our DL model has unique advantages. First, the imaging data could be stored and used repeatedly, in addition, the noninvasive deep learning method is more convenient than the pathological approach based on biological specimens. Secondly, we should be cognizant that deep learning models can produce study results more directly and quickly and improve the problem of being time-consuming and burdensome for busy clinicians compared with ROI-based analysis. This may be attributed to the automatic feature extraction based on deep learning models probably have the ability to capture additional differences within tumors [36, 37]. Moreover, we enrolled a higher sample size of over 1800 CRC patients, which met the requirement of millions of weights to train efficiently for CNN, and our model demonstrated superior performance to previous radiological models in terms of AUC values (0.74–0.82 for other models) [31, 38, 39]. In conclusion, it is suggested that the DL model based on CT images has the potential to stratify tumor MMR status with promising effectiveness prior to surgery, chemotherapy, or immunotherapy in our study.

Despite promising findings, our study has some limitations. First, the retrospective nature of this study inevitably leads to selection bias, and a prospective and multicenter study is required to confirm the impact of our model in the future so that it could serve better in clinical application. Second, although we had a favorable predictive performance in participants from an external institution, it did not reach a high level in the internal institution. We considered that no specific schemes were applied to deal with the parameter variations from different scanners. Third, we explored the DL model based on routine CT images rather than other imaging technologies, such as MRI and Dual-Energy computed tomography, which have been reported in previous studies [39, 40]. Actually, CT is the most suitable and routine examination method for colorectal cancer. Finally, since there is no specific definition of these extracted features of deep learning, their interpretability should further explore image encoding processes in the future.

## Conclusions

We have constructed and validated the fully automatic DL model derived from pre-therapeutic CT images, which can stratify MMR status in CRC, with superior performance. This method could provide a potential noninvasive tool to triage MMR status in CRC, thus further personalized medicine.

## Supplementary Information

**Additional file 1: Figure S1.** The flowchart of inclusion and exclusion criteria for eligible patients in the study.

**Additional file 2: Figure S2.** The ROC curves of Resnet101 and VGG-19.

**Additional file 3: Figure S3.** The ROC curves of DL model based on the CT images of X, Y and Z axis respectively and the Gaussian regression fusion model.

**Additional file 4: Figure S4.** The ROC curves of different Gaussian regression models.

**Additional file 5.** The representative CT and immunohistochemistry images of different MMR statuses.

**Additional file 6.** The results of subgroups analyses.

## Data Availability

The datasets used during this study are available from the corresponding author on reasonable request.

## Abbreviations

CRC: Colorectal cancer
dMMR: DNA mismatch repair deficient
MSI-H: Microsatellite instability-high
FDA: The United State Food and Drug Administration
NCCN: The National Comprehensive Cancer Network
CNN: Convolutional neural network
CEA: Carcinoembryonic antigen
CA199: Cancer antigen 199
CA125: Cancer antigen 125
CA153: Cancer antigen 153
CT: Computed Tomography
DL: Deep learning
PACS: The Picture Archiving and Communication System
ROI: Region of interest
GPR: Gaussian process regression
ROC: Receiver operating characteristic curve
AUC: The area under the receiver operating characteristic curve
PPV: Positive predictive value
NPV: Negative predictive value
