# Supplementary figures and images for: CT-based deep learning model for the prediction of DNA mismatch repair deficient colorectal cancer: a diagnostic study
Source: J Transl Med. 2023 Mar 22;21:214. doi: 10.1186/s12967-023-04023-8 (PMC10035255; doi:10.1186/s12967-023-04023-8)

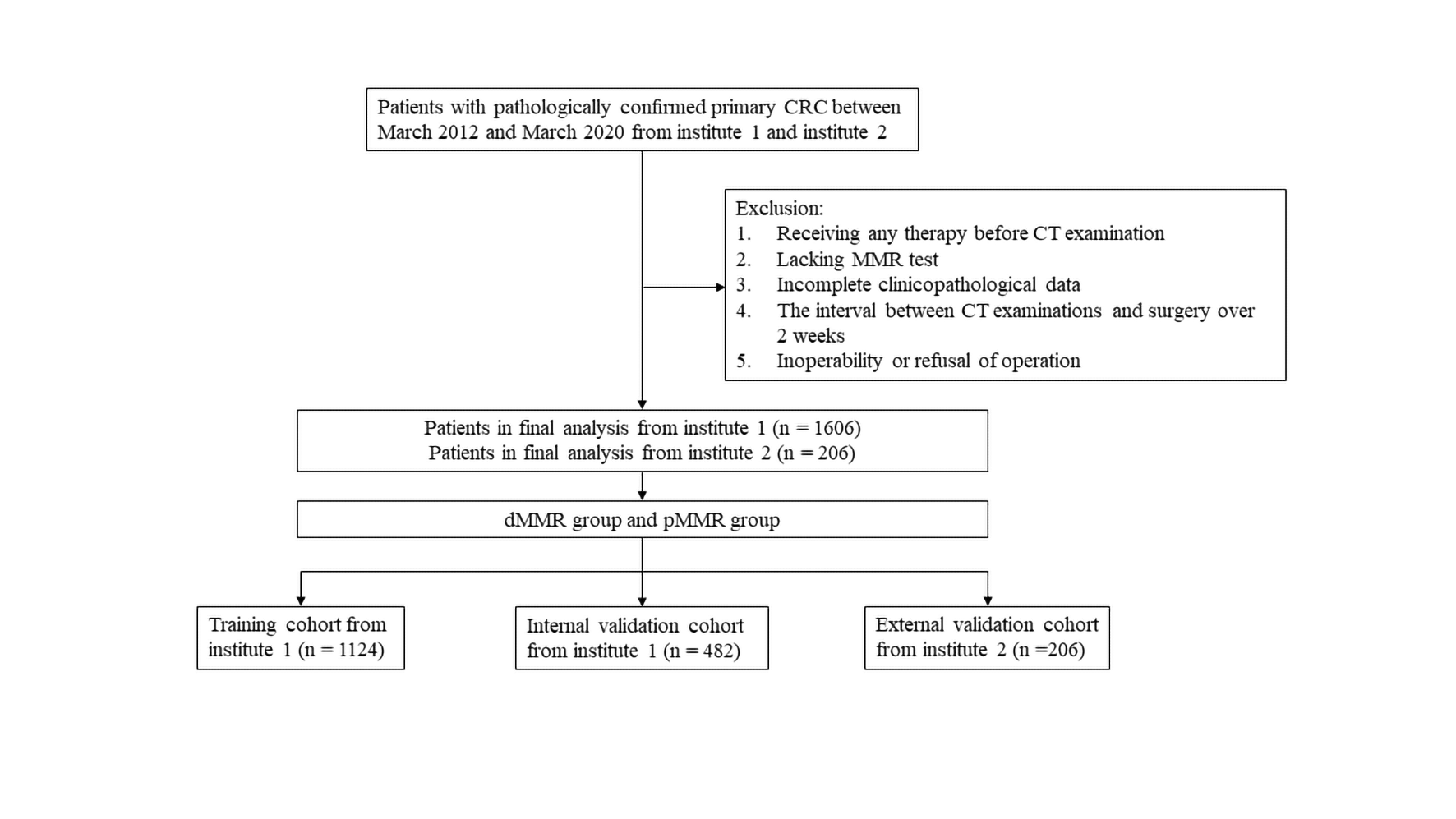

Supplement: Supplementary file 1 — Additional file 1: Figure S1. The flowchart of inclusion and exclusion criteria for eligible patients in the study. [file 12967_2023_4023_MOESM1_ESM.tif]

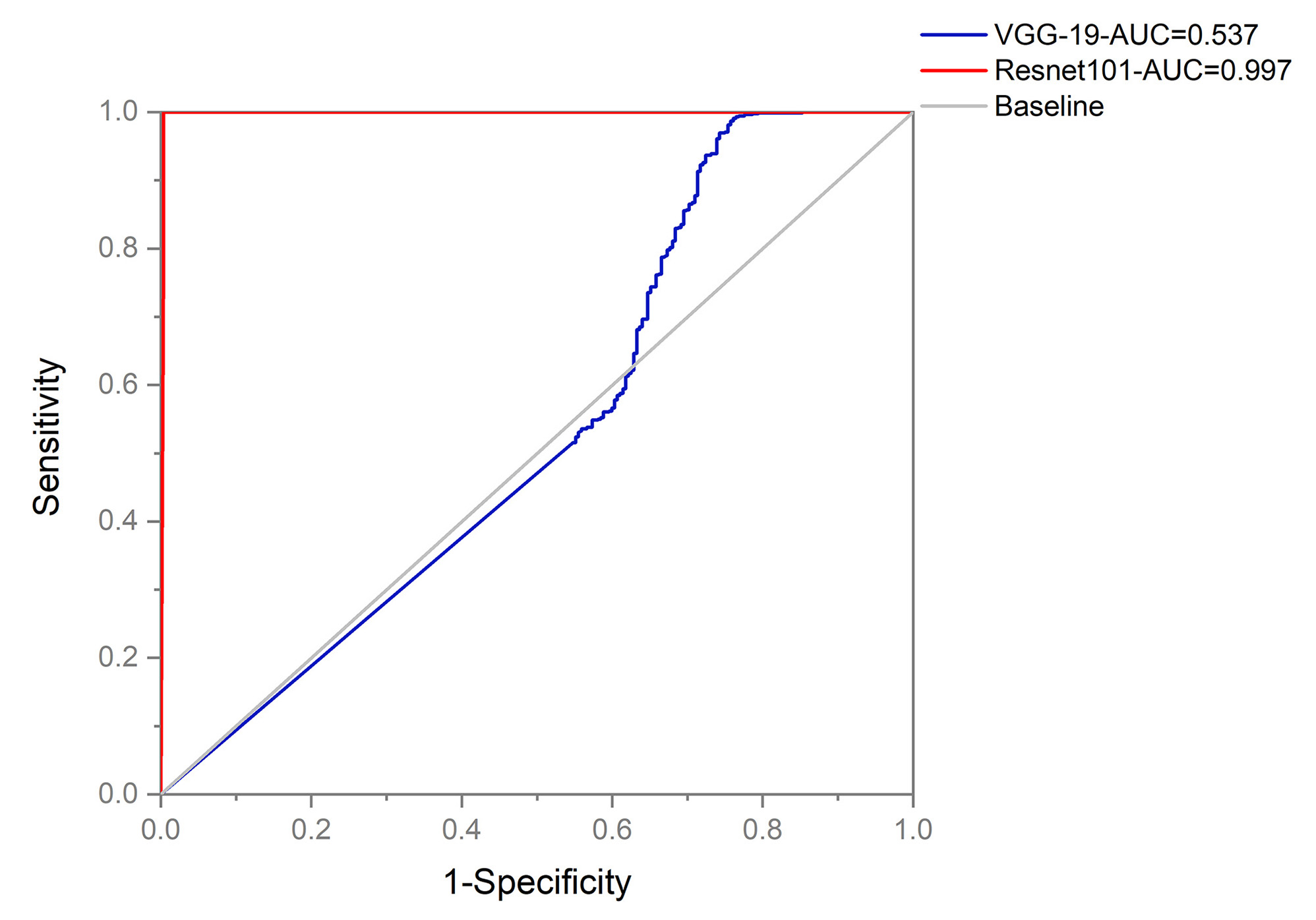

Supplement: Supplementary file 2 — Additional file 2: Figure S2. The ROC curves of Resnet101 and VGG-19. [file 12967_2023_4023_MOESM2_ESM.jpg]

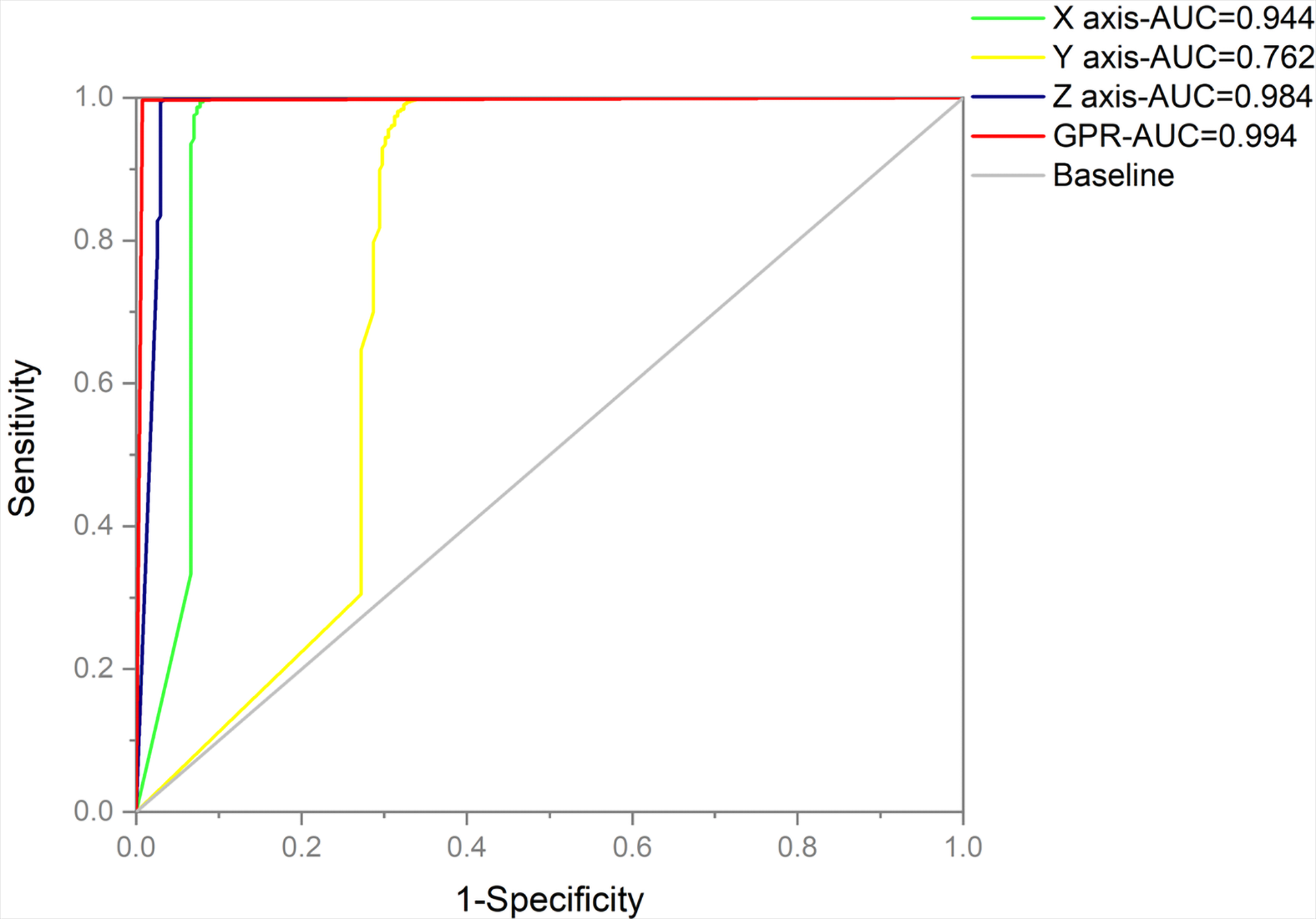

Supplement: Supplementary file 3 — Additional file 3: Figure S3. The ROC curves of DL model based on the CT images of X, Y and Z axis respectively and the Gaussian regression fusion model. [file 12967_2023_4023_MOESM3_ESM.tif]

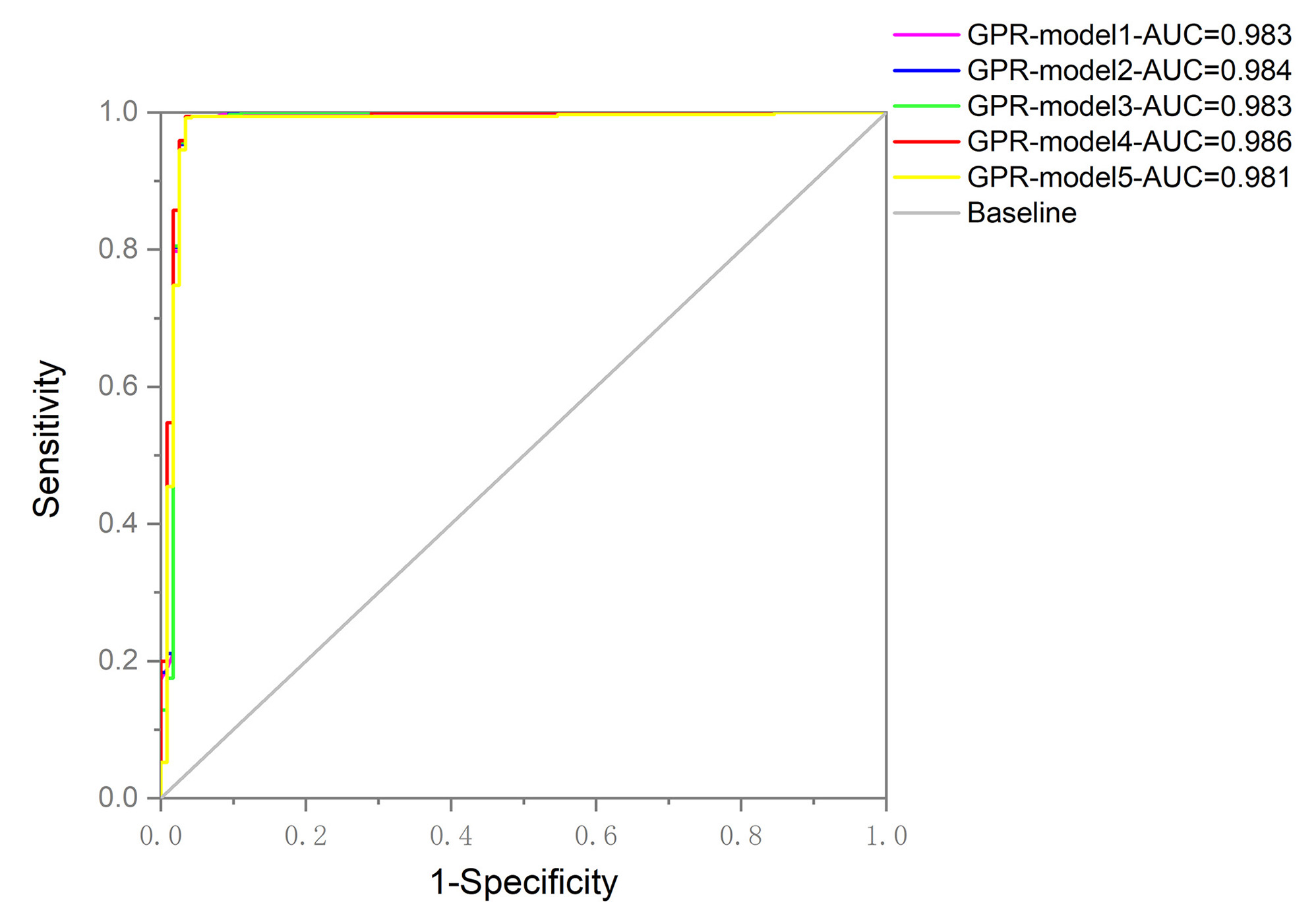

Supplement: Supplementary file 4 — Additional file 4: Figure S4. The ROC curves of different Gaussian regression models. [file 12967_2023_4023_MOESM4_ESM.jpg]
