# Supplementary material for: CT-based deep learning model for the prediction of DNA mismatch repair deficient colorectal cancer: a diagnostic study
Source: J Transl Med. 2023 Mar 22;21:214. doi: 10.1186/s12967-023-04023-8 (PMC10035255; doi:10.1186/s12967-023-04023-8)
Supplement: Supplementary file 5 — Additional file 5. The representative CT and immunohistochemistry images of different MMR statuses. [file 12967_2023_4023_MOESM5_ESM.pptx]

## Slide 1
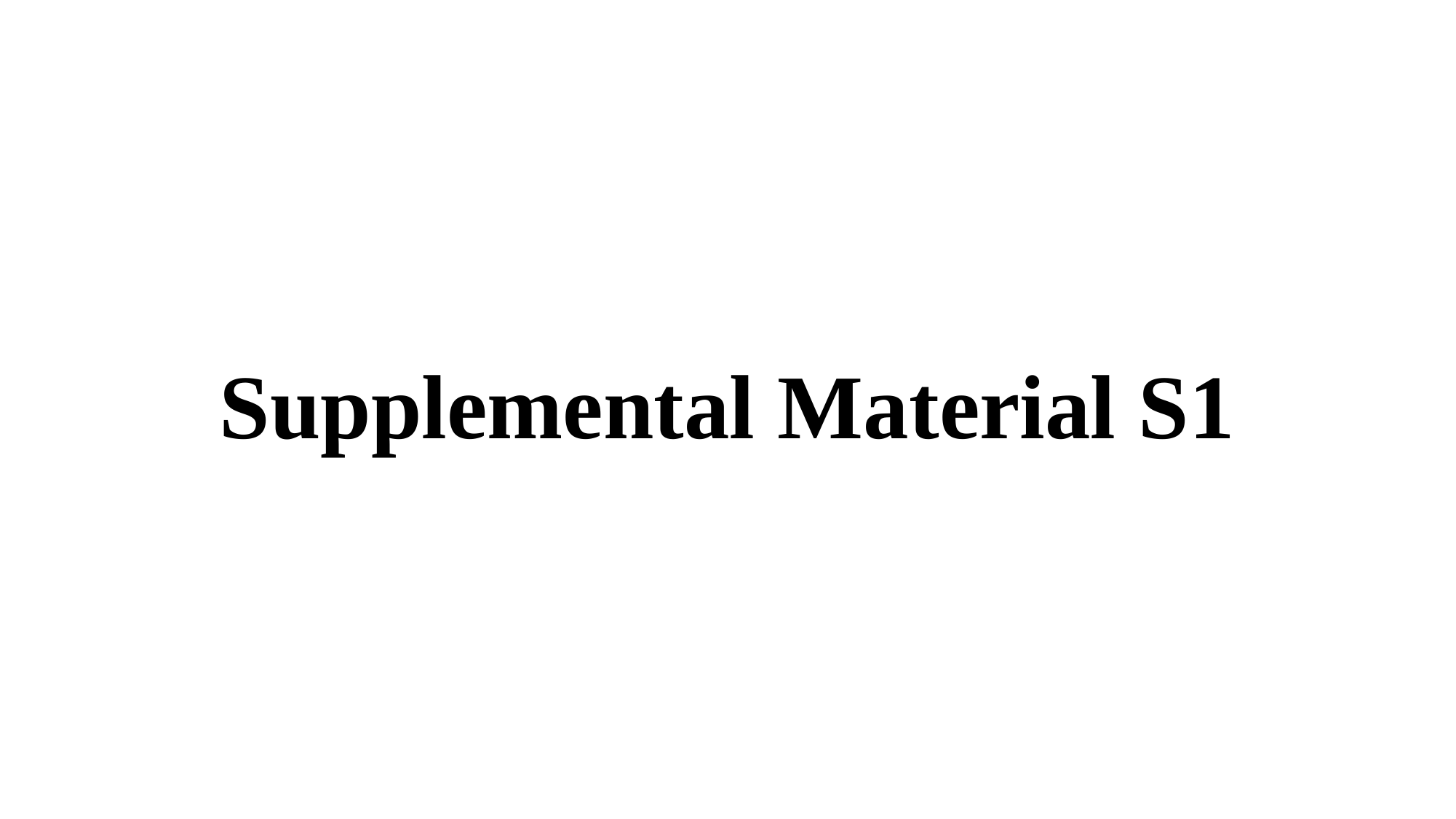

# Supplemental Material S1

## Slide 2
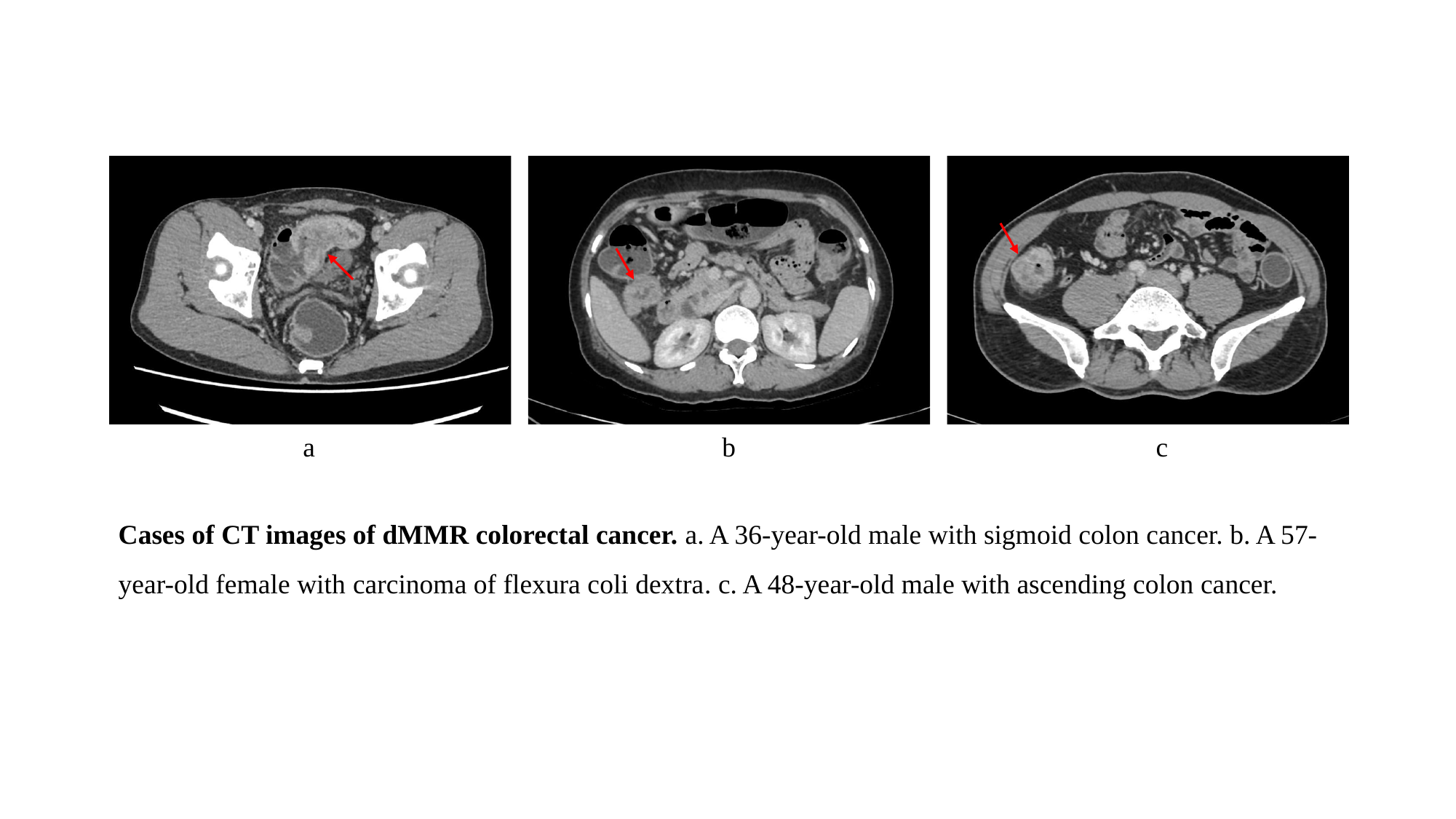

a
b
c
Cases of CT images of dMMR colorectal cancer. a. A 36-year-old male with sigmoid colon cancer. b. A 57-year-old female with carcinoma of flexura coli dextra. c. A 48-year-old male with ascending colon cancer.

## Slide 3
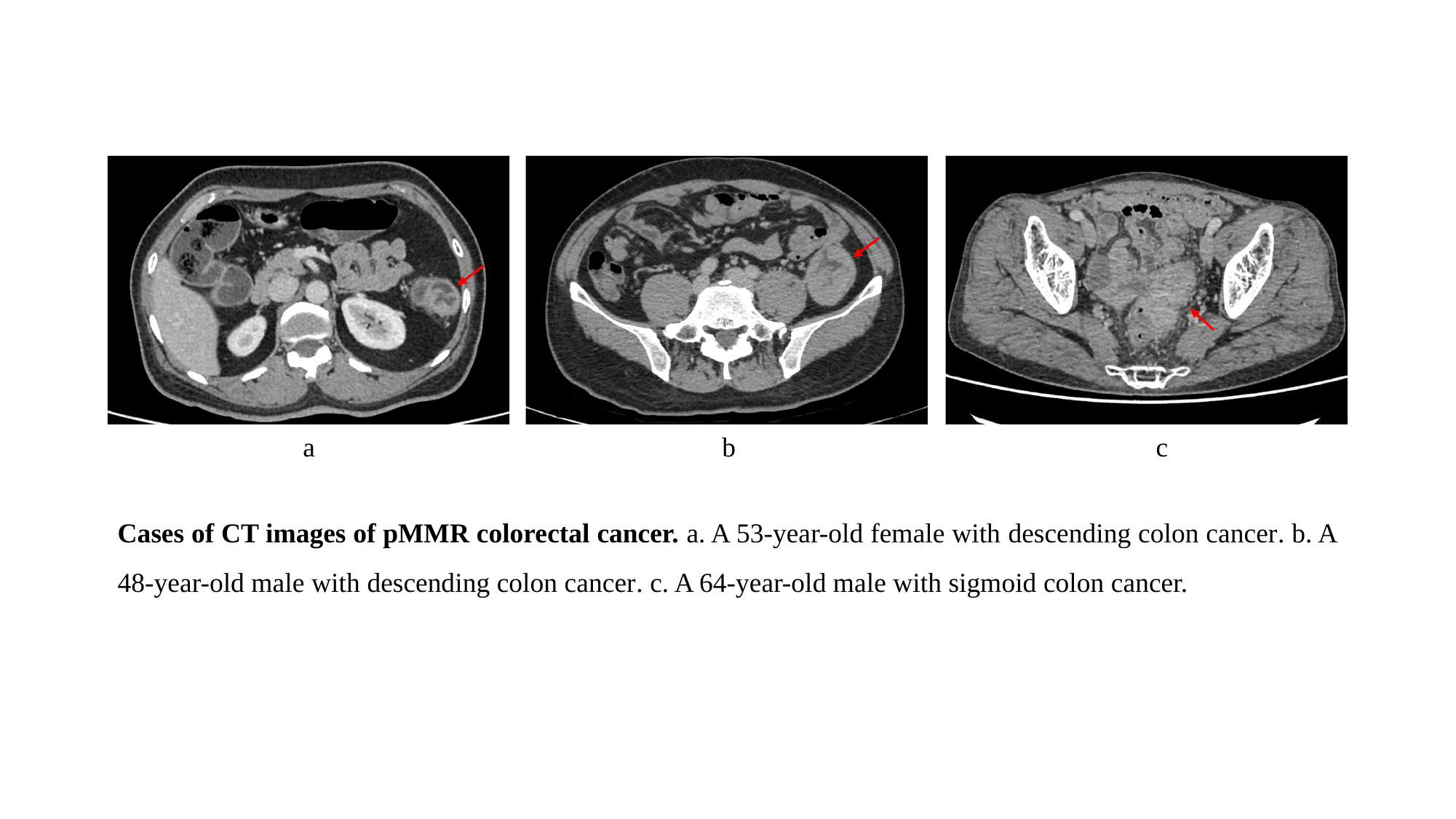

a
b
c
Cases of CT images of pMMR colorectal cancer. a. A 53-year-old female with descending colon cancer. b. A 48-year-old male with descending colon cancer. c. A 64-year-old male with sigmoid colon cancer.

## Slide 4
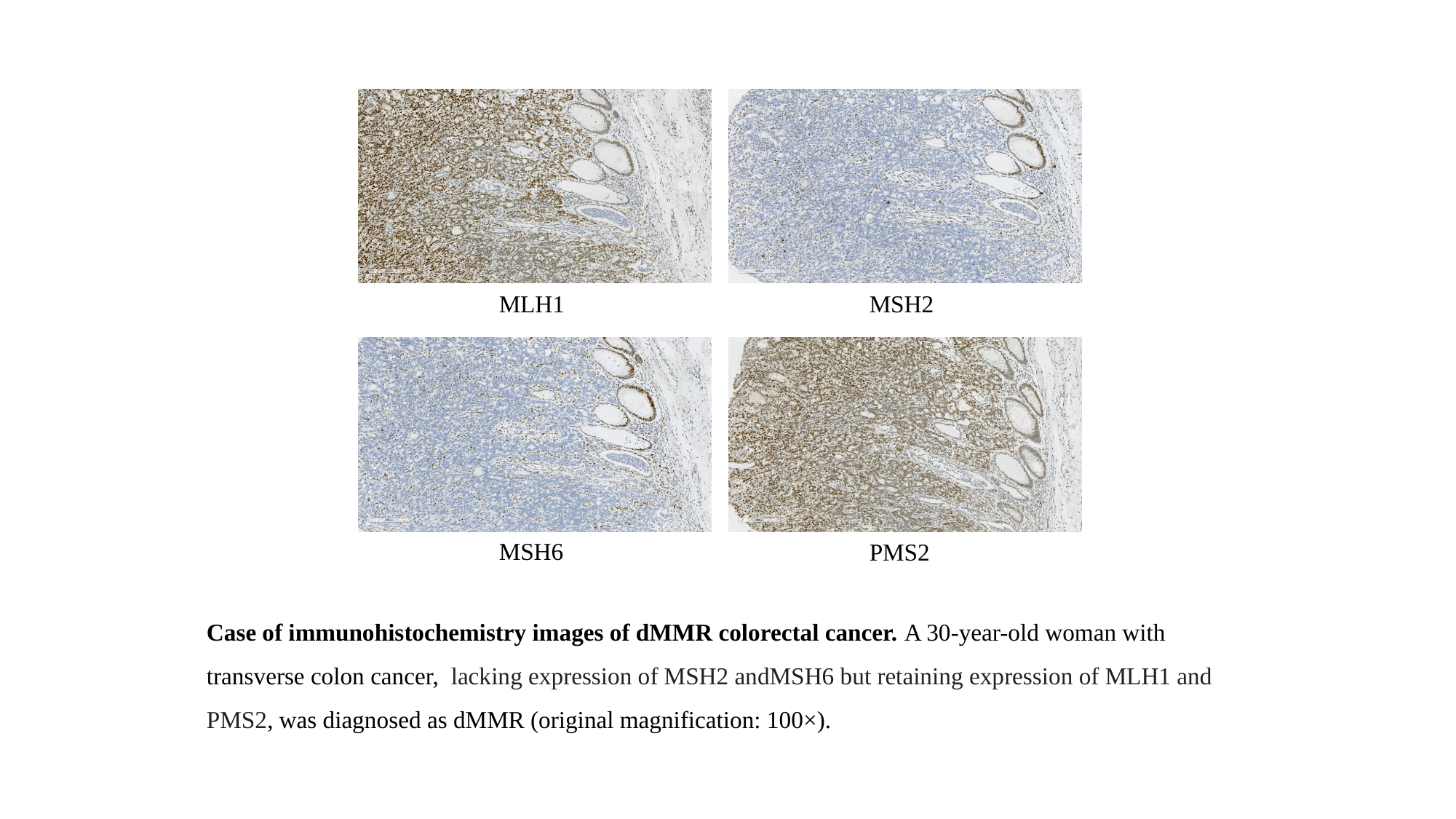

MLH1
MSH2
MSH6
PMS2
Case of immunohistochemistry images of dMMR colorectal cancer. A 30-year-old woman with transverse colon cancer,  lacking expression of MSH2 andMSH6 but retaining expression of MLH1 and PMS2, was diagnosed as dMMR (original magnification: 100×).

## Slide 5
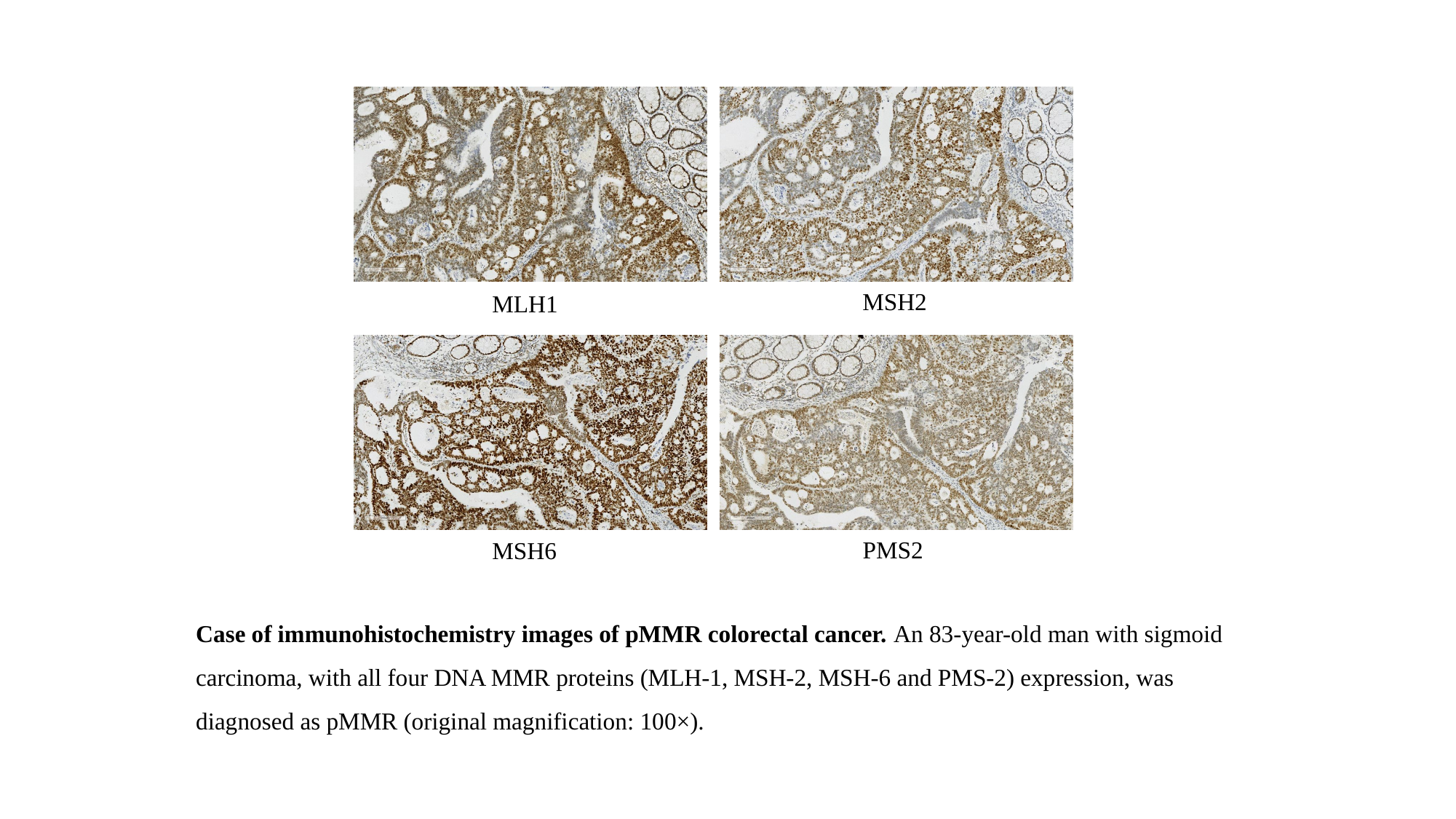

MSH2
MLH1
PMS2
MSH6
Case of immunohistochemistry images of pMMR colorectal cancer. An 83-year-old man with sigmoid carcinoma, with all four DNA MMR proteins (MLH-1, MSH-2, MSH-6 and PMS-2) expression, was diagnosed as pMMR (original magnification: 100×).
