# Supplementary material for: CT-based deep learning model for the prediction of DNA mismatch repair deficient colorectal cancer: a diagnostic study
Source: J Transl Med. 2023 Mar 22;21:214. doi: 10.1186/s12967-023-04023-8 (PMC10035255; doi:10.1186/s12967-023-04023-8)
Supplement: Supplementary file 6 — Additional file 6. The results of subgroups analyses. [file 12967_2023_4023_MOESM6_ESM.docx]

**Supplementary Material S2**

Subgroups analyses were performed in addition to main analysis in order to assess prediction performance in different subgroups based on the thickness of CT images, clinical T and N stages, gender, the longest diameter and location of tumor in participants enrolled from institution 1. These clinical parameters were reviewed and recorded from the medical record archives, and classified as follows. The thickness of CT images were divided into two groups (thick-layer＞3mm, thin-layer ≤ 3mm). We dichotomized the clinical T stage as T1-2 or T3-4, and the clinical N stage was classified as N0 (without metastatic lymph node), N1 (metastasis in one-three regional lymph nodes) or N2 (metastasis in four or more regional lymph nodes). In addition, the longest diameter (LD) and location of tumor were evaluated by interpreting the CT images, then divided into various groups respectively (LD < 3 or ≥ 3 cm, and location of tumor, the right or left). The subgroup analysis revealed that the MMRnet model show similar satisfying prediction performance in all groups, as presented in the following table.

| **Subgroups** | **AUC** | **95%CI** | **Sensitivity** | **Specificity** | **PPV** | **NPV** |
| --- | --- | --- | --- | --- | --- | --- |
| **Thickness of CT images** |  |  |  |  |  |  |
| Thin | 0.992 | 0.981-1.000 | 0.992 | 0.981 | 0.992 | 0.981 |
| Thick | 0.914 | 0.758-1.000 | 1.000 | 0.778 | 0.984 | 1.000 |
| **Gender** |  |  |  |  |  |  |
| Male | 0.981 | 0.955-1.000 | 0.991 | 0.953 | 0.987 | 0.968 |
| Femal | 0.994 | 0.982-1.000 | 1.000 | 0.981 | 0.993 | 1.000 |
| **T Stage** |  |  |  |  |  |  |
| 1-2 | 1.000 | - | 1.000 | 1.000 | 1.000 | 1.000 |
| 3-4 | 0.986 | 0.970-1.000 | 0.994 | 0.965 | 0.989 | 0.982 |
| **N Stage** |  |  |  |  |  |  |
| 0 | 0.949 | 0.860-1.000 | 0.978 | 0.941 | 0.989 | 0.889 |
| 1 | 0.990 | 0.976-1.000 | 1.000 | 0.955 | 0.986 | 1.000 |
| 2 | 1.000 | - | 1.000 | 1.000 | 1.000 | 1.000 |
| **LD** |  |  |  |  |  |  |
| ≥3cm | 0.959 | 0.896-1.000 | 0.974 | 0.960 | 0.987 | 0.923 |
| ＜3cm | 0.993 | 0.983-1.000 | 1.000 | 0.967 | 0.990 | 1.000 |
| **Primary tumor Location** |  |  |  |  |  |  |
| Left | 0.974 | 0.926-1.000 | 0.996 | 0.970 | 0.996 | 0.970 |
| Right | 0.991 | 0.979-1.000 | 0.992 | 0.964 | 0.975 | 0.988 |

Note. LD= the longest diameter, AUC= area under the curve, PPV= positive predictive value, NPV= negative predictive value.
